# Effects of Alkaline Solutions on the Structure and Function of Influenza A Virus

**DOI:** 10.3390/v16101636

**Published:** 2024-10-19

**Authors:** Manato Seguchi, Seiji Yamaguchi, Mamoru Tanaka, Yukihiro Mori, Masato Tsurudome, Morihiro Ito

**Affiliations:** 1 Graduate School of Life and Health Sciences, Chubu University, 1200 Matsumoto-cho, Kasugai-shi 487-8501, Aichi, Japan; rb24801-4974@sti.chubu.ac.jp (M.S.); sy-esi@isc.chubu.ac.jp (S.Y.); tsuru@isc.chubu.ac.jp (M.T.); 2 Support for Pioneering Research Initiated by the Next Generation (SPRING), Chubu University, 1200 Matsumoto-cho, Kasugai-shi 487-8501, Japan; 3 Department of Biomedical Sciences, College of Life and Health Science, Chubu University, 1200 Matsumoto-cho, Kasugai-shi 487-8501, Aichi, Japan; 4 Department of Food and Nutritional Sciences, College of Bioscience and Biotechnology, Chubu University, 1200 Matsumoto-cho, Kasugai-shi 487-8501, Aichi, Japan; m-tanaka@isc.chubu.ac.jp; 5 Department of Nursing, College of Life and Health Science, Chubu University, 1200 Matsumoto-cho, Kasugai-shi 487-8501, Aichi, Japan; moriyuki0821@isc.chubu.ac.jp; 6 Department of Lifelong Sports and Health Sciences, College of Life and Health Sciences, Chubu University, 1200 Matsumoto-cho, Kasugai-shi 487-8501, Aichi, Japan

**Keywords:** influenza A virus, alkaline condition, hemagglutinin, hydrolysis

## Abstract

Influenza A virus (IAV) infection contributes to high annual morbidity and mortality, thus necessitating measures aimed at protecting against the disease. Alcohol-based disinfectants are commonly used to inactivate IAV, but they have several undesirable properties. In search of other means which would inactivate IAV, we focused on the effect of alkaline solutions on IAV. We found the viral infectivity remarkably decreased with treatment of an alkaline solution at pH 12.0 for 1 min, where destruction of the viral spikes was observed using an electron microscope. A more detailed examination revealed that the infectivity of IAV was remarkedly reduced by brief treatment with the alkaline solution at pH 11.75 or above, most likely due to the degradation of viral hemagglutinin protein. These results show that at a high pH, the haemagglutinin protein is degraded, resulting in very rapid inactivation of IAV.

## 1. Introduction

Since the outbreak of the influenza A virus (IAV) pandemic in 1918, which caused 50 million deaths, IAV has remained a global public health concern [1,2]. Approximately 290,000 to 650,000 people die from influenza each year, typically resulting in seasonal epidemics [3]. IAVs are negative-sense single-stranded RNA viruses with an envelope belonging to the Orthomyxoviridae family. The eight RNA genomes coated with the NP protein are called nucleocapsids, which are enclosed within the envelope. The envelope harbors hemagglutinin (HA) and neuraminidase as viral spike proteins [4,5]. The HA precursor, HA0, is cleaved into disulfide-linked HA1 and HA2 subunits (namely HA1 + HA2) by trypsin-like host cell proteases such as TMPRSS2 during the course of proliferation. Infection by IAV begins when HA1 binds to sialoconjugate receptors on the cell surface [6,7]. It then enters the cell by endocytosis and low pH-induced conformational changes in HA2 result in membrane fusion between the viral envelope and endosomal membrane, which enables the inner nucleocapsid to enter the cytoplasm and initiate transcription and replication [6,8]. Because red blood cells have sialoconjugate receptors, IAV can exhibit so-called HA activity, i.e., cross-link and agglutinate red blood cells when mixed with them [9]. When infected with IAV, humans present with a variety of symptoms [1,10]. In addition, it can cause severe illness, potentially resulting in death [10,11]. Despite various efforts to prevent IAV infection, the disease persists unabated [12]. The contributing factor is that sometimes viruses from other hosts pass to humans by changing their receptor specificity [13]. In this case, they easily spread around the world since people do not have antibodies to the new variant of the virus.

It is crucial to take precautions to prevent contraction of the disease [14], especially with the use of alcohol disinfectants, which are used for hand disinfection and the surface disinfection of objects that are touched by hands and are mainly employed to prevent infections caused by enveloped viruses. Rachidi et al. reported that hand disinfection with alcohol disinfectants, which is primarily used as an infection control measure, caused dermatological problems in approximately 47% of humans and psychological problems due to stress and discomfort in approximately 11% of people [15]. Therefore, a safer and more reliable infection prevention method must be identified.

Microorganisms can survive within a certain pH range, and changes in environmental conditions due to pH make it impossible for microorganisms to survive, making control effective. As for IAV, structural changes in and functions of HA proteins have been extensively studied, especially in the context of pH changes [16]. Takeda et al. reported that the treatment of IAV with an acidic aqueous solution at a pH of three for 10 min resulted in a loss of infectivity and HA activity [17]. Furthermore, Baatartsogt et al. reported that acidic aqueous solutions induced morphological changes in the virus and caused the dysfunction of HA proteins, resulting in the loss of infectivity of IAV [18]. Importantly, in this context, the premature activation of HA protein by acid treatment seems to play a major role in inactivating IAV, as reported by Tosheva et al. [19]. On the other hand, IAV is also inactivated by alkaline pH, but the underlying mechanism has not been studied [20,21,22].

In this study, we investigated the effects of alkaline solutions on the infectivity and structure of IAV, revealing the molecular basis of IAV inactivation by alkaline solutions.

## 2. Materials and Methods

### 2.1. Preparation of Alkaline Solutions

Alkaline solutions of pH 10.0 to pH 12.0 were prepared using phosphate-buffered saline (PBS: 155.17 mM NaCl, 1.06 mM KH_2_PO_4_, 2.97 mM Na_2_HPO_4_) and 5.0 N NaOH (FUJIFILM Wako Pure Chemical Corporation, Osaka, Japan) in saline; eventually, the NaOH concentration in the alkaline solution (pH 12.0) was calculated to be 8.6 × 10^−6^ M.

### 2.2. Cells and Viruses

Madin–Darby bovine kidney (MDCK; #CCL-34, ATCC, Manassas, VA, USA) cells were cultured in growth medium, namely Eagle’s minimum essential medium (EMEM; #051-07615, FUJIFILM Wako Pure Chemical Corporation) supplemented with fetal bovine serum (FBS; #F7524, Sigma-Aldrich, St. Lois, MO, USA). Influenza A virus H1N1 A/Puerto Rico/8/34 strain (PR-8; #VR-95, ATCC) was propagated in MDCK cells and recovered 72 h after infection and stored at 80 °C until use. Viral infection was measured by a plaque assay using MDCK cells, as described below.

### 2.3. Plaque Assay

A total of 50 μL of virus solution (approximately 1.0 × 10^7^ PFU/mL) was mixed with 450 μL alkaline solution in TPX tubes (Sumitomo Bakelite Co., Ltd., Tokyo, Japan). After leaving for 1 min at 25 °C, the reaction was stopped by adding 4.5 mL SCDLP medium (#395-00265, FUJIFILM Wako Pure Chemical Corporation) and serial ten-fold dilution was carried out using MEM. Then, 100 μL of each diluted mixture was added onto MDCK cell monolayers grown in 12-well culture plates, kept at 37 °C for 1 h, and the cells were stratified with 0.5% agarose in MEM containing acetylated trypsin from bovine pancreas ((#T6763, Sigma-Aldrich) after removal of the mixture. After incubating for 72 h at 37 °C, the agar-covered monolayers were overlayed with 1 mL of 10% neutral buffered formalin solution at RT for 90 min. After removal of the agar, the monolayers were stained with methylene blue and the virus titer (PFU/mL) was determined.

### 2.4. Transmission Electron Microscope Assay

Alkaline solutions (450 μL each) were dispensed in TPX tubes. Then, 50 μL of virus solution, approximately 1.0 × 10^7^ PFU/mL, was added to and mixed with the alkaline solutions in each tube. After leaving for 1 min at 25 °C, the mixture was stirred well and 10 μL of the stirred virus solution (500 μL) was placed on a high-resolution carbon substrate STEM Cu 100P (HRC-C10; #10-1013, Okenshoji, Tokyo, Japan) with an integrated carbon deposition film and copper grid. After 10 min, the water on each sample on the grid was drained and the samples were treated with a 2% 12-tungstophosphoric acid solution (12 Tungsto (Ⅵ) phosphoric acid n-hydrate; #162-02432, FUJIFILM Wako Pure Chemical Corporation) for 30 s at 25 °C. Virus samples were observed using a field emission electron microscope (#JEM-2100F, Japan Electron Optics Laboratory, Tokyo, Japan).

### 2.5. Sodium Dodecyl Sulfate–Polyacrylamide Gel Electrophoresis

A total of 50 μL of virus solution (approximately 1.0 × 10^7^ PFU/mL) was mixed with 450 μL alkaline solution in TPX tubes. After being left for 1 min at 25 °C, the mixture was stirred well and was added with the sample buffer (Tris 312.50 mM, SDS 346.76 mM, sucrose 730.35 mM, bromophenol blue [BPB] 0.15 mM) containing or not containing 2-mercaptoethanol [2-ME]) at a ratio of 4:1, of which 24 μL was subjected to sodium dodecyl sulfate–polyacrylamide gel electrophoresis (SDS–PAGE) under reducing or non-reducing conditions using a 10%–20% polyacrylamide gel (e-PAGEL ATTO, Taito-ku, Tokyo, Japan). It was then used for Western blotting or silver staining.

### 2.6. Western Blotting

After subjected to SDS-PAGE, the IAV proteins in the gel were electrically blotted onto a polyvinylidene fluoride (PVDF) membrane (AmershamTM HybondTM P PVDF 0.2; #10600021, Cytiva, Tokyo, Japan) and the membrane was blocked overnight at 4 °C with 2% ECLTM Prime Blocking Reagent (#RPN418, Cytiva). The membrane was then treated for 1 h at 25 °C with rabbit anti-IAV HA2 monoclonal antibody [23] (IAV HA/HA Antibody, Rabbit MAb; #86001-RM01, Sino Biological, Inc., Beijing, China) or rabbit anti-IAV nucleoprotein (NP) monoclonal antibody (IAV nucleoprotein antibody; #GTX636247, Gene Tex, Inc., Irvine, CA, USA) as primary antibodies. Subsequently, the membrane was treated for 1 h at 25 °C with horseradish peroxidase (HRP)-conjugated anti-rabbit IgG (#HAF008, R&D Systems, Inc., Minneapolis, MN, USA) as the secondary antibody. Finally, the HA and NP protein bands were visualized by chemiluminescence with the aid of iBright FL1500 Imaging Systems (Thermo Fisher Scientific) using an ECL^TM^ Prime Western Blotting System (#RPN2232, Cytiva).

### 2.7. Silver Staining

After being subjected to SDS-PAGE as described above, the IAV proteins in the gel were stained with EzStain Silver (#AE-1360, ATTO) according to the manufacturer’s protocol. Gels were shaken with a fixing solution (40 mL purified water + 40 mL methanol + 10 mL acetic acid + 1 mL S-1 solution) for 10 min, then washed three times with 100 mL purified water for 10 min. After washing, the cells were treated with staining solution (100 mL purified water + 1 mL S-2 solution) for 5 min; the staining solution was discarded and washed with 100 mL purified water for 30 s. Subsequently, the cells were washed with 100 mL of a coloring solution (200 mL purified water + 1 mL S-3 solution + 1 mL S-4 solution) for 30 s, replaced with a new coloring solution, and shaken for 7 min. The color was then stopped by mixing with a reaction stop solution (100 mL purified water + 1 mL acetic acid) for 10 min and photographed with iBright FL1500 Imaging Systems (Thermo Fisher Scientific).

### 2.8. HA Activity

A total of 50 μL of virus solution (approximately 1.0 × 10^7^ PFU/mL) was mixed with 450 μL alkaline solution in TPX tubes. After it had been left to stand for 1 min at 25 °C, the mixture was stirred well and serial two-fold dilution of the mixture was performed using PBS and 50 μL of each diluted mixture was mixed with 50 μL guinea pig red blood cell solution (#035-00012, Japan Bio Serum, Hiroshima, Japan) in a 96-well round-bottom plate and kept on ice for 120 min; then, the HA titer was calculated.

## 3. Results

### 3.1. Antiviral Effect of Alkaline Solutions on IAV

**Figure 1:**
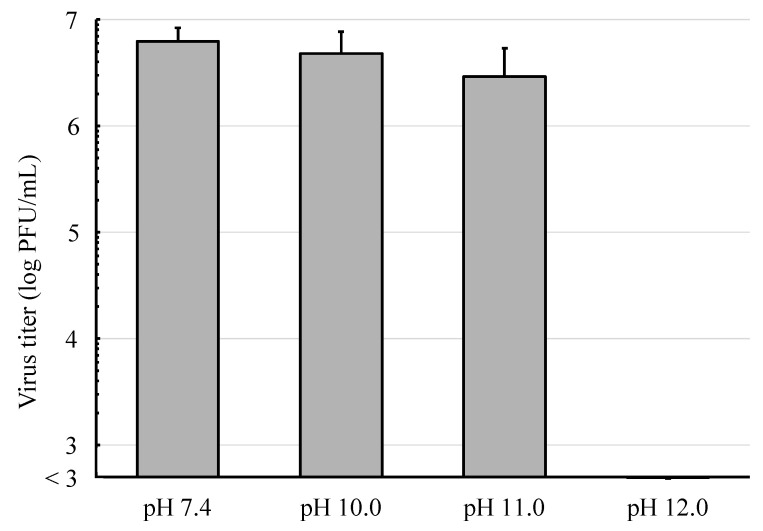
MDCK cells were infected with IAV, which had been treated with alkaline solutions for 1 min and incubated for 72 h. Then, the viral infectivity was evaluated by a plaque assay and the results of quadruple samples were presented as mean ± standard deviation.

The infectivity of IAV was not significantly affected after treatment with alkaline solutions at pH 10.0 or 11.0 (Figure 1). By contrast, treatment with the alkaline solution at pH 12.0 reduced the infectivity by more than three orders of magnitude compared to the control.

### 3.2. Effect of Alkaline Solutions on Viral Proteins

**Figure 2:**
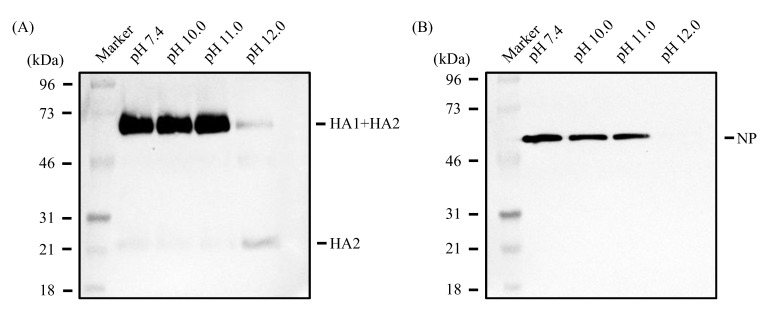
IAVs were treated with alkaline solution and subjected to SDS-PAGE under non-reducing conditions followed by Western blot under non-reducing conditions, as described in the Materials and Methods. Data are representative of three independent experiments. The HA protein was detected by a rabbit monoclonal antibody specific for the HA protein (**A**) and the NP protein was detected by a rabbit monoclonal antibody specific for the nucleoprotein protein (**B**).

When analyzed by Western blotting under non-reducing conditions (Figure 2A,B), viral proteins, HA (HA1 + HA2), and NP could be detected after treatment with the alkaline solution at pH 10.0 or 11.0 as efficiently as those at pH 7.4 (control). By contrast, these proteins became barely detectable after treatment with the alkaline solution at pH 12.0; interestingly, however, the amount of HA2 subunits apparently increased compared with the control (Figure 2A).

### 3.3. Effect of Alkaline Solutions on Viral Morphology

**Figure 3:**
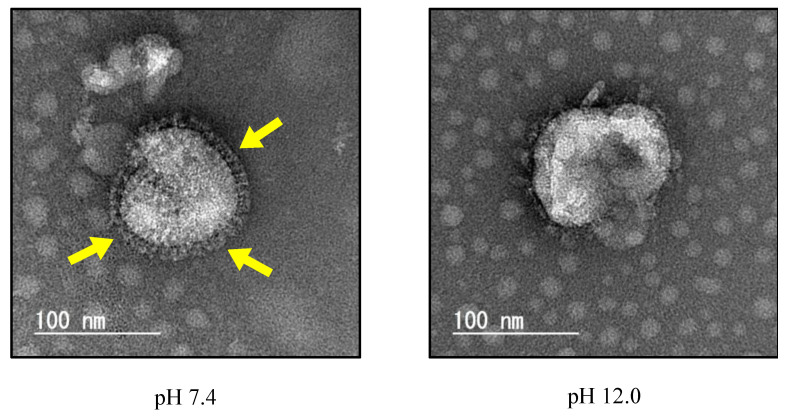
IAV particles treated with alkaline solutions were negatively stained and images were obtained using transmission electron microscopy. Yellow indicates viral spikes. Data are representative of two independent experiments.

The effect of alkaline solutions on the morphology of IAV particles is shown in Figure 3. Surface spikes on IAV particles at pH 7.4 were clearly visible. By contrast, such spikes could not clearly be observed after treatment with the alkaline solution at pH 12.0. In order to minimize possible mechanical damage to the IAV particles, the alkaline-treated viral particles were not further concentrated.

### 3.4. Effect of Alkaline Solutions on IAV Infectivity

**Figure 4:**
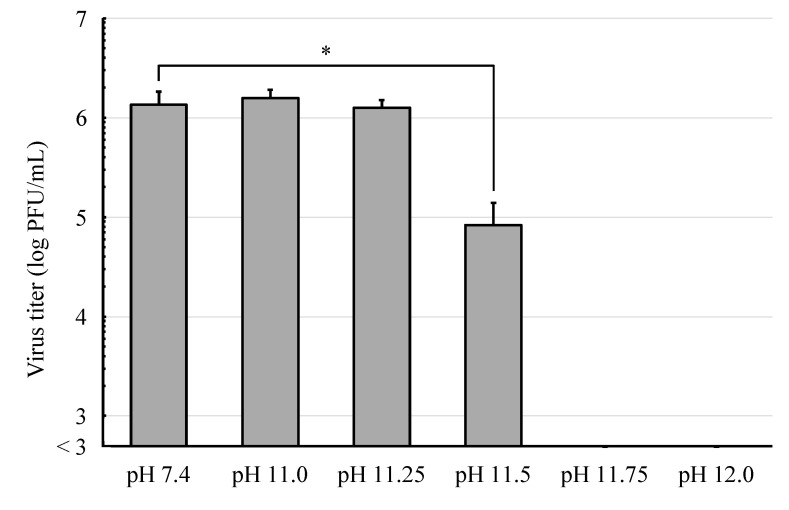
MDCK cells were infected with IAV which had been treated with more detailed alkaline solutions for 1 min (*n* = 4) and incubated for 72 h, and the viral infectivity was evaluated by a plaque assay. The results are presented as mean ± standard deviation. * *p* < 0.01.

**Figure 5:**
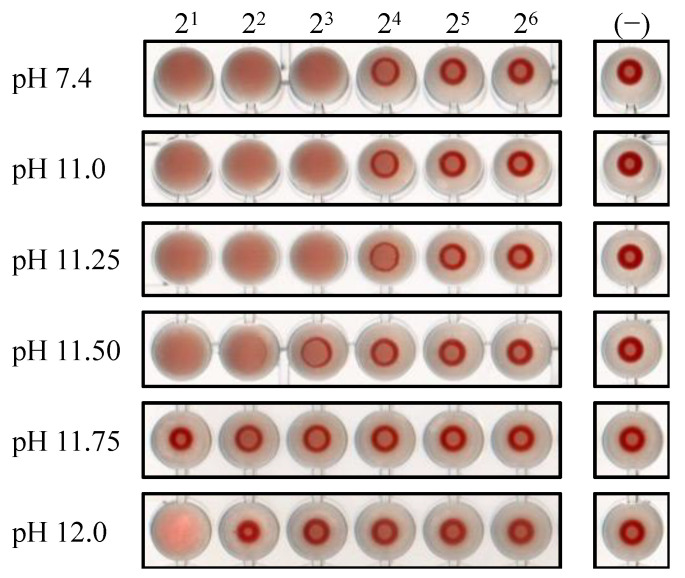
IAVs treated with alkaline solutions for 1 min were subjected to serial two-fold dilution in PBS, mixed with guinea pig erythrocytes in a round-bottom 96-well plate, and incubated at 4 °C for 120 min. The graphical results are representative of three independent experiments.

As described so far, the infectivity and protein stability of IAV was clearly affected by treatment of IAV with the alkaline solution at pH 12.0 but not by the alkaline solution at pH 11.0. To further analyze these findings, we examined the effect of alkaline solutions at pH 11.25, 11.5, or 11.75 on the IAV infectivity (Figure 4). Interestingly, the infectivity of IAV was also not affected by the alkaline solution at pH 11.25, while it was reduced by approximately one order of magnitude by the alkaline solution at pH 11.5 compared to the control. Notably, treatment of IAV with alkaline solutions at pH 11.75 or pH 12.0 reduced the infectivity by more than three orders of magnitude. Consistently, the alkaline solutions at pH 11.0 or pH 11.25 did not affect the viral HA activity, while those at pH 11.75 and pH 12.0 caused a noticeable reduction in the viral HA activity (Figure 5). The leftmost well in the lane at pH 12.0 was observed under a microscope and it was confirmed that the guinea pig red blood cells had been hemolyzed.

### 3.5. Denaturation of IAV Proteins by Alkaline Solutions

**Figure 6:**
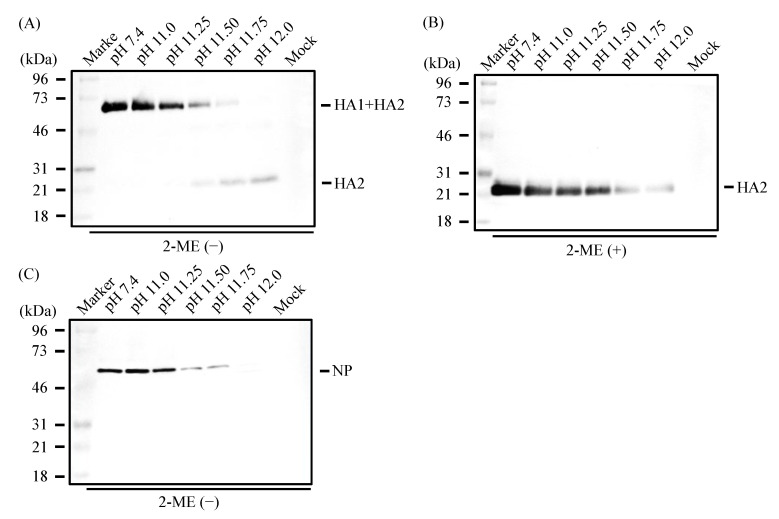
IAVs were treated with alkaline solutions and subjected to SDS-PAGE under non-reducing (**A**,**C**) or reducing (**B**) conditions followed by Western blot, as described in the Materials and Methods. Data are representative of three independent experiments. The HA protein was detected by a rabbit monoclonal antibody specific for the HA protein (**A**,**B**) and the NP protein was detected by a rabbit monoclonal antibody specific for the nucleoprotein protein (**C**).

**Figure 7:**
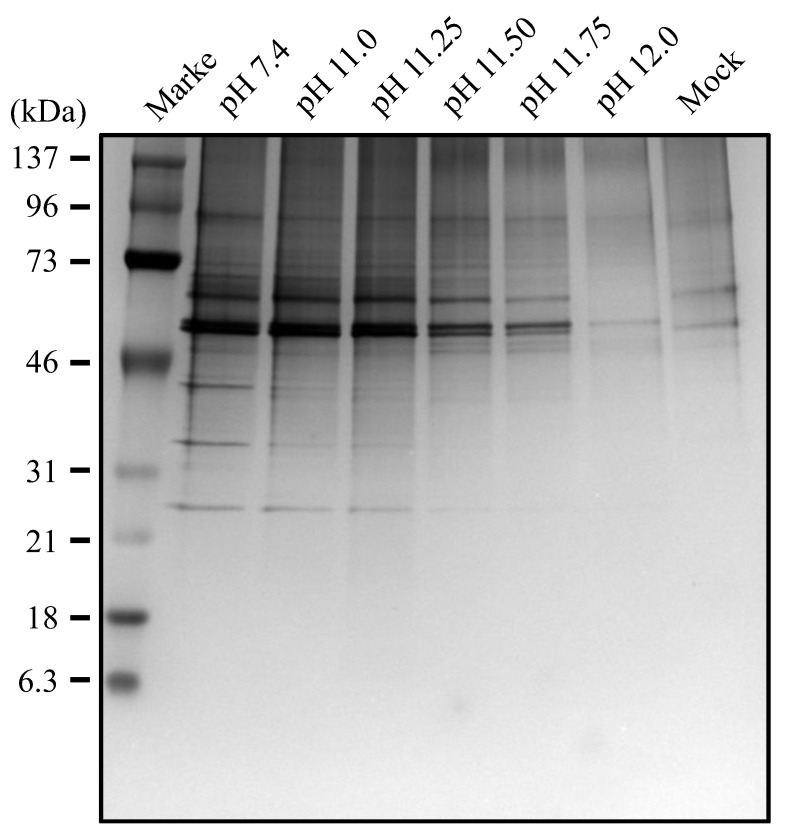
IAVs were treated with alkaline solutions and subjected to SDS-PAGE under non-reducing conditions followed by silver staining, as described in the Section 2.

As described above (Figure 2), the HA protein was detected after treatment with the alkaline solution at pH 11.0 as efficiently as the control but became barely detectable after treatment of IAV with the alkaline solution at pH 12.0. Intriguingly, however, the amount of HA2 subunits apparently increased compared with the control. To further investigate these phenomena, we treated IAV with alkaline solutions at pH 11.25, 11.5, and 11.75, and performed an analysis by Western blotting under non-reducing conditions (Figure 6A). We found that as the pH of the alkaline treatment solution was raised, the amount of HA2 subunits increased with a concomitant loss of HA1 + HA2, suggesting that the inter-subunit disulfide bond in HA was disrupted under alkaline conditions. Intriguingly, however, as revealed by SDS-PAGE under reducing conditions (Figure 6B), the amount of HA2 subunits, which reflects the total amount of the HA protein in the sample, decreased as the treatment pH was raised. Similarly, the amount of NP protein also decreased as the pH of the treating alkaline solution increased (Figure 6C). Being consistent with these findings, any protein contained in the sample became hardly detectable by treatment with the alkaline solution at pH 12.0, indicating that these proteins had undergone efficient hydrolysis (Figure 7).

## 4. Discussion

In this study, we found that the infectivity and receptor-binding activity of IAV was remarkedly affected by brief treatment with alkaline solutions at pH 11.75 or above.

Consistent with our findings, it was reported that treatment of various H7N3 avian influenza virus strains with alkaline solutions at pH ≥ 10 for ≥24 h results in a reduction in viral erythrocyte aggregation activity [20]. Moreover, the H5N1 avian influenza virus strain has been reported to have decreased erythrocyte aggregation activity when treated with an alkaline solution at pH ≥ 11 for more than 6 h [21].

The result of the Western bot has revealed that when IAV was treated with an alkaline solution at pH 11.5 or higher, the HA1 + HA2 band disappeared with the generation of the HA2 band, suggesting that alkaline hydrolysis cleaved the inter-subunit disulfide bond between HA1 and HA2 (i.e., releasing the receptor-binding subunit HA1 from the virus particle) and incited further degradation of the HA protein. It has already been reported that aqueous NaOH solutions can cleave disulfide bonds [24] and that at high alkaline pH, the extreme charge repulsive forces of the polypeptide chain cause the protein to unfold, exposing hydrophobic groups and free sulfhydryl (-SH) groups [25]. Moreover, high-alkaline conditions may induce changes in the tertiary and quaternary structure and composition of proteins by alkali-induced hydrolysis [26,27]. Since a study on SARS coronavirus has also demonstrated a decrease in the spike protein activity and suppression of infectivity at a pH ≥12 after 1 h [28], alkaline solutions appear to have the potential to denature spike proteins of envelope viruses. Consistent with these findings, our present study has also shown that the total amount of HA protein seemed to decrease as the pH of the alkaline solution was raised, most likely suggesting that the HA may had been denatured by the alkaline solution and lost its antigenicity. Whether alkaline solutions have a similar effect on other enveloped viruses has not yet been determined. Further research also needs to be directed toward the effects of alkaline solutions on non-enveloped viruses, which have not been investigated thus far.

On the other hand, hydroxyl ions have been reported to induce the peroxidation of lipids, leading to the destruction of phospholipids [29]. Our present finding that the NP protein was degraded by alkaline solutions strongly suggests that the phospholipid bilayers of the viral envelope was disrupted and the hydroxyl ions reached viral nucleocapsids, resulting in the destruction of the NP protein.

Our study has revealed, as described above, that alkaline conditions affect the structure and function of IAV, resulting in a remarkable loss of viral infectivity at pH 11.75 or above. Therefore, although it is well known that highly alkaline conditions are harmful and can cause physiological disorders, we anticipate that such alkaline solutions could be useful as an IAV inactivating agent. Of note, in this context, a buffering effect may occur on human skin, i.e., a previous study by Zhai et al. revealed that the pH of skin which had been treated with 0.025 N NaOH decreased from 8.3 ± 0.8 to 6.2 ± 0.6 within 30 min [30]. The NaOH concentration we employed in this study (8.6 × 10^−6^ M) to create the alkaline solution with pH 12 was much lower than that used by Zhai et al. [30]. Nakashima et al. also reported that the pH value of the skin surface was 8.84 ± 1.17 one minute after applying an alkaline aqueous solution with a pH of 12.39 ± 0.03 to a human hand, and that this solution had no adverse effects on human skin [31]. In addition, the results of rabbit eye toxicity tests using OECD Chemical Substances Testing Guideline no. 405 “Acute Eye Irritation/Corrosion” [32] showed no adverse effects [31]. Therefore, it seems very likely that the risk to the human body posed by alkaline solutions is extremely low due to the buffering effect of human skin. Moreover, previous studies have suggested that even alkalis have no effect on human skin depending on the conditions [33]. It is worth pointing out in this context that alkaline solutions are already used in humans, such as for the treatment of root canal infection [29], burns [34,35], and atopic dermatitis [36]. Thus, although there may be some caveats, we believe that alkaline solutions at pH 11.75 or pH 12.0 have great potential as a new way to prevent infections.

## 5. Conclusions

In this study, we have studied the effects of alkaline solutions on the structure and function of Influenza A virus. Our current findings indicate that alkaline solutions at pH 11.75 or above effectively inactivate IAV by hydrolyzing the viral spike protein, hemagglutinin.

## Data Availability

Not applicable.
